# Preparation of size-controlled LiCoPO_4_ particles by membrane emulsification using anodic porous alumina and their application as cathode active materials for Li-ion secondary batteries

**DOI:** 10.1039/d3ra01035j

**Published:** 2023-06-01

**Authors:** Takashi Yanagishita, Raraka Otomo, Hideki Masuda

**Affiliations:** a Department of Applied Chemistry, Tokyo Metropolitan University 1-1 Minamiosawa Hachioji Tokyo 192-0397 Japan yanagish@tmu.ac.jp

## Abstract

Membrane emulsification using anodic porous alumina is an effective method for preparing monodisperse droplets with controlled sizes. In this study, membrane emulsification using anodic porous alumina was applied to the preparation of size-controlled particles composed of composite metal oxides. To obtain size-controlled composite metal oxide particles, membrane emulsification was performed using an aqueous solution containing a water-soluble monomer and metal salts as a dispersed phase. After the membrane emulsification, composite metal oxide particles were obtained by solidifying the droplets in a continuous phase and subsequent heat treatment. Here, as a demonstration of this process, the fabrication of size-controlled LiCoPO_4_ particles, which are considered high-potential cathode active materials for Li-ion secondary batteries (LIBs), was investigated. The application of the obtained LiCoPO_4_ particles as cathode active materials for LIBs was also investigated. The results of this study showed that LiCoPO_4_ particles with controlled sizes could be fabricated on the basis of this process and that their cathode properties could be improved by optimizing the heat treatment conditions and particle sizes. According to this process, size-controlled particles composed of various metal oxides can be fabricated by changing the metal salt in the dispersed phase, and the resulting size-controlled particles are expected to be applied not only as cathode active materials for LIBs but also as components of various functional devices.

## Abstract 2

Preparation process for LiCoPO_4_ particles by membrane emulsification using anodic porous alumina.

## Graphical abstract

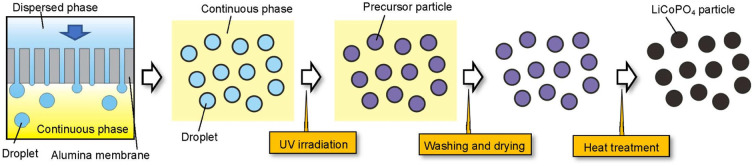

## Introduction

Fine particles composed of metals, semiconductors, and polymers are useful building blocks for fabricating various functional devices, such as electrical, optical, and biological devices.^1–10^ Various methods have been reported for the preparation of particles with size in the submicron to nanometer scales, including the reductive deposition of metal ions in a liquid phase, chemical vapor deposition (CVD), and spray drying.^11–14^ Among them, membrane emulsification has been attracting considerable attention as an effective method for preparing monodisperse particles with controlled sizes. In membrane emulsification, a dispersed phase is excluded into a continuous phase through an emulsification membrane with uniform-sized pores to form monodisperse droplets.^15^ Droplet size can be controlled by changing the pore size of the emulsification membrane. When producing monodisperse solidified particles, a dispersed phase that can be solidified by a subsequent process is used for emulsification. We have studied membrane emulsification using anodic porous alumina for the preparation of monodisperse particles with sizes in the submicron to nanometer scale.^16–18^ Anodic porous alumina is a honeycomb-shaped porous film with uniform-sized cylindrical pores, and the pore size can be controlled precisely from single-digit nanometers to micron scales by controlling the fabrication conditions.^19,20^ Therefore, anodic porous alumina is a promising material as a porous membrane for membrane emulsification to produce fine monodisperse particles with controlled sizes in the nanometer range. In our previous studies, we found that monodisperse polymer particles could be prepared by membrane emulsification using a monomer as a dispersed phase.^21^ It has also been reported that monodisperse particles composed of various metal oxides can be fabricated by membrane emulsification using a sol solution containing primary nanoparticles of metal oxides as a dispersed phase.^22^ However, the materials of fine particles that can be produced by membrane emulsification are limited because the solidification of droplets in a continuous phase is necessary to obtain monodisperse solidified particles. To expand the application field of membrane emulsification using anodic porous alumina, it is important to establish a versatile process that can form fine particles of a wide variety of materials.

In this report, the combination of a water-soluble monomer (acrylamide monomer) and metal salts is introduced to enable the preparation of particles of a wide variety of materials by membrane emulsification using anodic porous alumina. In this method, precursor particles composed of a polymer and metal salts were prepared through the polymerization and solidification of droplets in a continuous phase. With the subsequent heat treatment of the resulting precursor particles, the production of size-controlled fine particles composed of various metal oxides and composite oxides is expected. The polymer used as the matrix acts as a precursor for carbon. It is also expected that metal oxide particles containing carbon can be fabricated through heat treatment. Here, as a demonstration, we showed the preparation of size-controlled LiCoPO_4_ particles, which are considered high-potential cathode active materials for Li-ion secondary batteries (LIBs). LiCoPO_4_ has a high redox potential of 4.8 V, excellent cycle characteristics due to small changes in crystal structure caused by the insertion and desorption of Li ions, and high thermal stability due to its high thermal decomposition temperature.^23^ However, because of its low ionic and electronic conductivities, much research has been conducted to reduce the diffusion distance of Li ions by decreasing the particle size and coating the surfaces of fine particles with carbon to increase electronic conductivity.^24–26^ By using the process reported here, we can expect that smaller particles can be prepared by reducing the pore size of the porous alumina membrane, and composite particles composed of LiCoPO_4_ and carbon can also be formed by optimizing the heat treatment conditions. In this study, in addition to the preparation of size-controlled LiCoPO_4_ particles by membrane emulsification using anodic porous alumina, the evaluation of their cathode properties was also studied.

## Experimental section

### Preparation of emulsification membranes

**Fig. 1:**
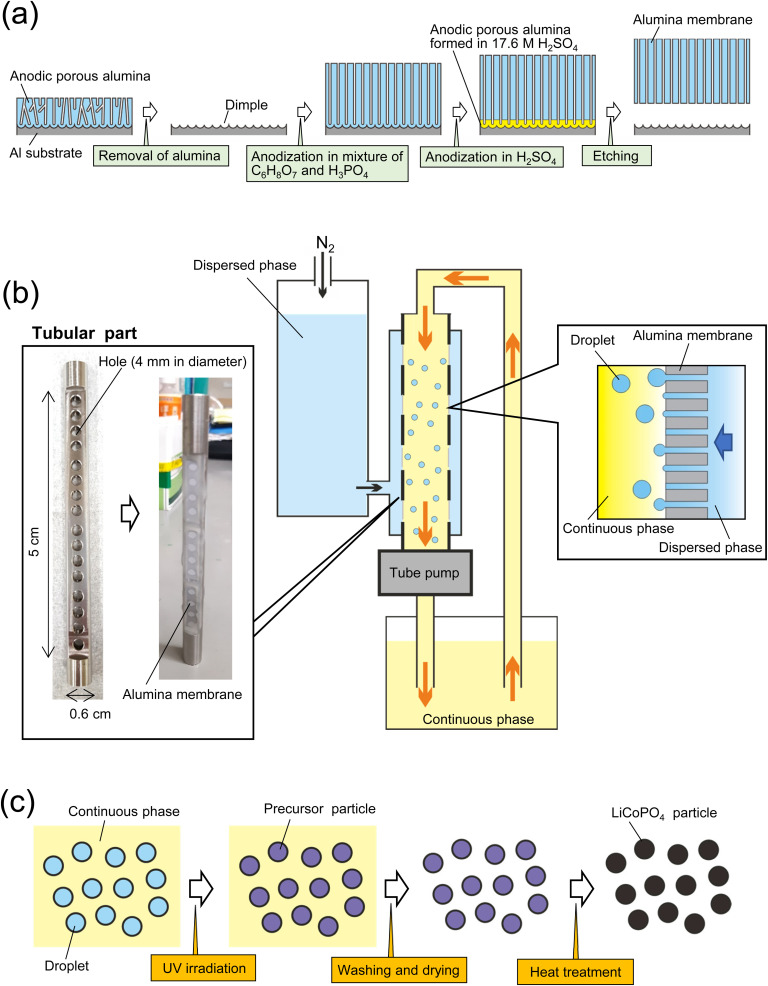
(a) Schematic of preparation process for anodic porous alumina membrane used as emulsification membrane. (b) Experimental setup for membrane emulsification using anodic porous alumina. (c) Schematic of preparation process for LiCoPO_4_ particles using emulsion droplets obtained by membrane emulsification.

Fig. 1(a) shows a schematic of the fabrication process for an alumina through-hole membrane used for membrane emulsification. An Al sheet (9 × 0.6 cm^2^) was electrochemically polished in a mixture of 20 vol% perchloric acid and 80 vol% ethanol at 0 °C under a constant current density of 0.1 A cm^−1^ for 2 min. The electropolished Al sheet was anodized in a mixture of 0.1 M oxalic acid and 0.25 M phosphoric acid at 0 °C under a constant voltage of 160 V for 3 h. After the anodization, the sample was immersed in a mixture of 1.8 wt% chromic acid and 6 wt% phosphoric acid at 70 °C for 2 h to dissolve the oxide film formed by the first anodization, obtaining an Al sheet with a dimple array on the surface. Since each dimple serves as a starting point for pore formation in the subsequent anodization, anodic porous alumina with an improved pore arrangement can be obtained. The Al sheet with the dimple array was anodized again for 65 h under the same conditions as the first anodization to form an oxide film with a thickness of 100 μm. To obtain an alumina through-hole membrane from the anodized sample, the oxide film was detached by the previously reported two-layer anodization method using concentrated sulfuric acid.^27,28^ The Al sheet with the anodized film on the surface was anodized in 17.6 M sulfuric acid at 0 °C under a constant voltage of 160 V for 150 min. This process resulted in the formation of an alumina layer containing high concentrations of sulfate ions, which is extremely soluble compared with normal anodic oxide films, under the oxide film formed by the previous anodization. The alumina layer formed in concentrated sulfuric acid was dissolved selectively in a mixture of chromic acid and phosphoric acid at 70 °C for 10 min to detach the alumina through-hole membrane from the Al substrate. The pore size of the alumina membrane was controlled by wet etching using 10 wt% phosphoric acid at 30 °C.

### Formation of LiCoPO_4_ particles by membrane emulsification

Fig. 1(b) shows a schematic of the process of preparing size-controlled LiCoPO_4_ particles by membrane emulsification using anodic porous alumina. For preparing size-controlled water droplets in an oil phase by membrane emulsification, a hydrophobic membrane must be used to prevent the wetting and spreading of the aqueous dispersed phase on the emulsified membrane surface. Since the surface of the alumina membrane obtained by Al anodization is hydrophilic, it was hydrophobically treated with tetradecylphosphonic acid in advance. To hydrophobize the alumina membrane, samples were immersed in a 40 g of ethanol solution containing 56 mg of tetradecylphosphonic acid at 50 °C for 20 h.^29^ All alumina membranes were then washed with ethanol and heat-treated at 100 °C for 10 min. The hydrophobically treated alumina membrane was attached to a tubular part, which has 16 holes with a diameter of 4 mm, with epoxy resin and set in a membrane emulsification device (KH-125, SPG Technology Co., Ltd., Japan). For membrane emulsification, an aqueous solution containing 17.4 wt% C_6_H_8_O_7_·H_2_O, 5.4 wt% LiH_2_PO_4_, 13 wt% (CH_3_COO)Co·H_2_O, 20 wt% IRGACURE 2959, and 2.5 wt% ammonia solution (28–30% in purity)was used. Kerosene solution containing surfactants, 2 wt% span 80 and 1 wt% tetraglycerin ester (CR-310, Sakamoto Yakuhin Kogyo Co., Japan), was used as a continuous phase. The continuous phase was circulated by a tube pump, whereas the dispersed phase was extruded through the pores of the alumina membrane into the continuous phase by pressurizing with N_2_ gas. The extrusion pressure during membrane emulsification was adjusted at 100 kPa. After 3 h of membrane emulsification, the resulting emulsion was irradiated with UV light for 10 h while stirring to polymerize the acrylamide monomer to obtain precursor particles, as shown in Fig. 1(c). After curing the droplets in the continuous phase, the precursor particles were collected by centrifugation and washed by two cycles of dilution with hexane and centrifugation. The precursor particles were dried and heat-treated in the air at temperatures ranging from 400 to 700 °C to obtain LiCoPO_4_ particles. During the heating process using an electric furnace, the temperature was increased at a rate of 5 °C min^−1^. After reaching the set temperature, the precursor particles were cooled without hold time at the set temperature. The size and shape of the obtained particles were determined by scanning electron microscopy (SEM; JSM-7500F, JEOL) and image processing software (Mac-View, Mountech Co., Ltd., Japan). The crystal structures of the obtained particles were evaluated by X-ray diffraction (XRD; RINT2000, Rigaku). A Raman spectrometer (NSR 4100, JASCO) and a thermogravimetric differential thermal analyzer (STA7300, Hitachi) were used to evaluate the carbon in the obtained particles.

### Formation of LiCoPO_4_ particles by starring emulsification

The LiCoPO_4_ particles with an average size of 1600 nm were prepared under the same conditions using the same raw materials, except for the emulsification method. The emulsion droplets were obtained by stirring the dispersed phase and continuous phase at 1000 rpm using a magnetic stirrer for 10 min. The precursor particles were obtained by UV irradiation, and LiCoPO_4_ particles were formed by heat treatment at 500 °C.

### Evaluation of electrochemical properties

The electrochemical properties of the obtained LiCoPO_4_ particles were evaluated using 2032 coin-type cells. The cathode was composed of LiCoPO_4_ particles, a conductive agent (acetylene black), and a polymer binder (polyvinylidene difluoride, PVDF) in a weight ratio of 75 : 15 : 10. These materials were suspended in *N*-methyl pyrrolidone and applied to the surface of Al foil using a doctor blade. The Al foil with the composite materials was dried in a vacuum oven at 110 °C for 5 h. The composite electrode was punched into a circular shape of 9 mm diameter. The resulting composite cathode, separator (Celgard #2400), Li foil anode, and electrolyte were assembled to form a 2032 coin-type cell. The electrolyte was a mixture of 50 vol% ethylene carbonate and 50 vol% diethylene carbonate containing 1 M LiPF_6_. Galvanostatic charge–discharge tests of the cell were performed at rates of 0.1 and 0.5C (1C = 167 mA g^−1^) in a potential range of 3.0 to 5.0 V at 30 °C.

## Results and discussion

### Formation of size-controlled LiCoPO_4_ particles by membrane emulsification

**Fig. 2:**
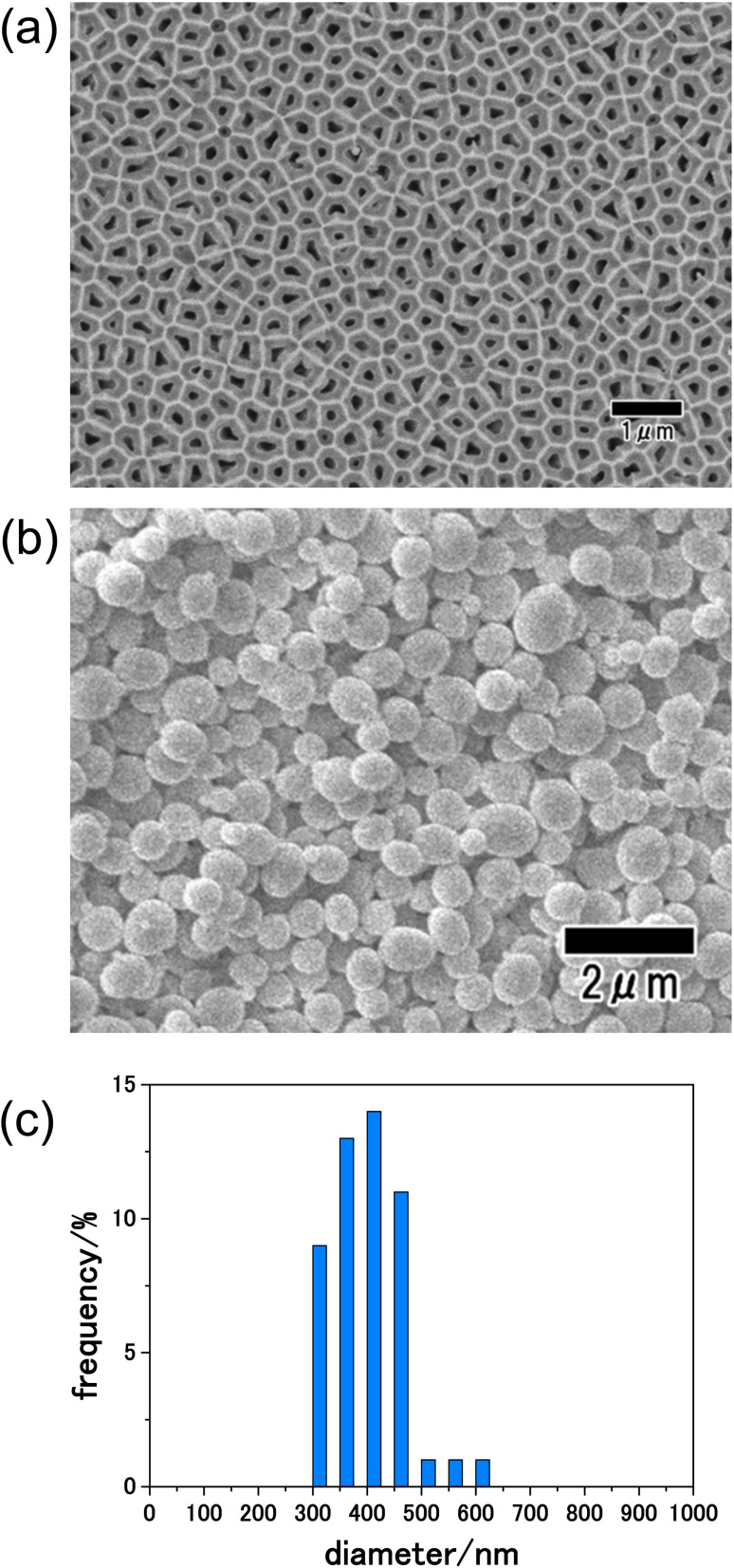
(a) SEM image of anodic porous alumina membrane used for membrane emulsification. (b) SEM images of precursor particles obtained by membrane emulsification using anodic porous alumina. (c) Size distribution histogram of precursor particles.

Fig. 2(a) shows a SEM image of the anodic porous alumina membrane used as an emulsification membrane. In this study, an alumina membrane with a thickness of 100 μm was used as the emulsification membrane to increase the mechanical strength of the membrane and to suppress membrane rupture during emulsification. Under the preparation conditions for the anodic porous alumina membrane used in this study, a long period of anodization is required to achieve a film thickness of 100 μm. The back surface of the alumina membrane was used as the emulsification surface during membrane emulsification because the pore size on the front surface of the membrane was increased by etching during the long period of anodization. The diameters of 300 pores on the back surface of the resulting membrane were measured from SEM images, and the average diameter was calculated to be 190 nm. Fig. 2(b) shows a SEM image of precursor particles obtained by the present process. From the SEM image shown in this figure, spherical particles with relatively uniform sizes were observed. Fig. 2(c) shows the size distribution histogram of the resulting particles prepared by measuring the diameters of 300 particles from SEM images. The obtained precursor particles had a relatively narrow diameter distribution, with an average diameter of 410 nm. The anodic porous alumina with a thickness of 100 μm used as the emulsification film in this study has high mechanical strength and did not fracture even when the emulsification pressure was 300 kPa.

**Fig. 3:**
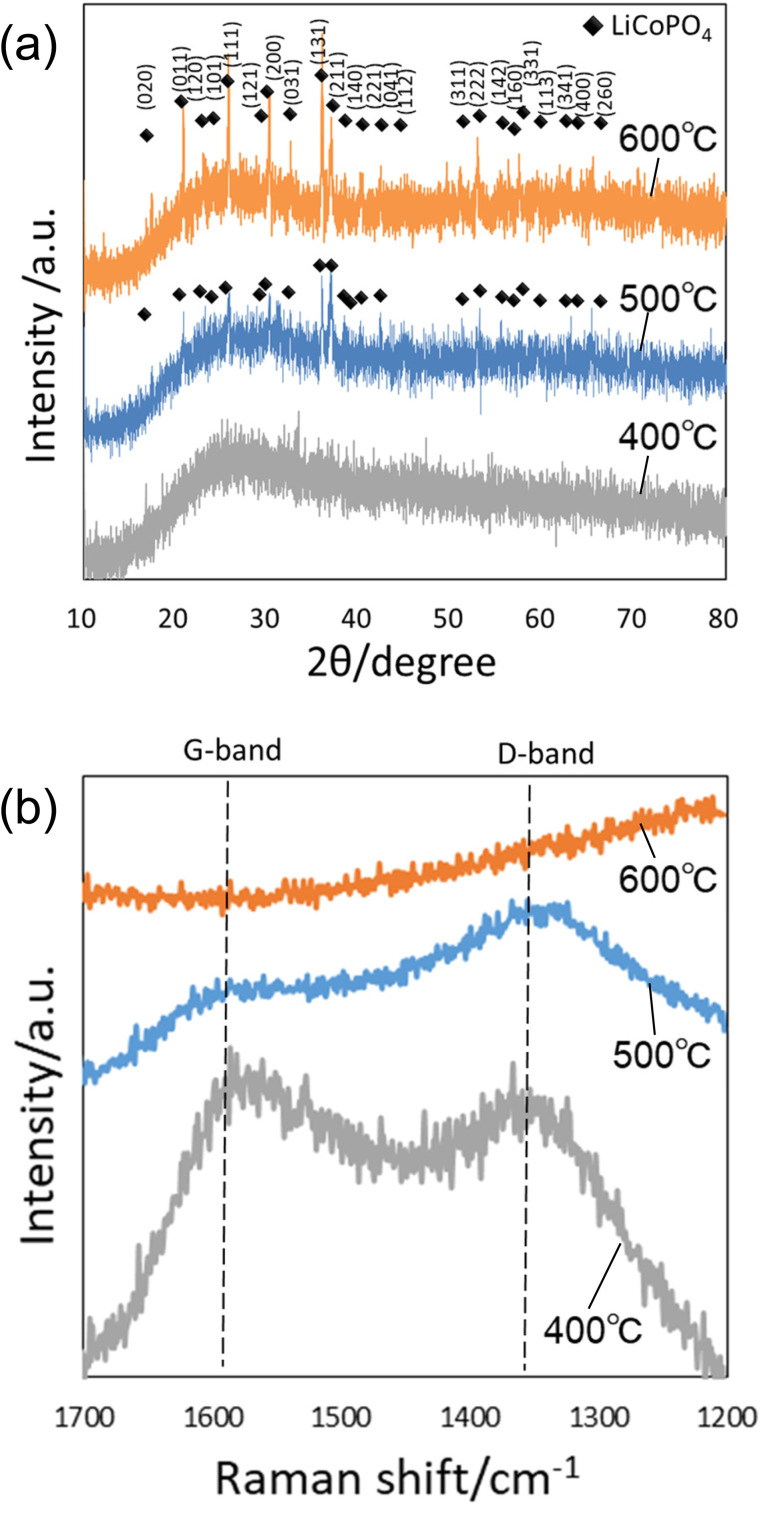
(a) XRD patterns and (b) Raman spectra of particles after heat treatment at 400, 500, and 600 °C.

Fig. 3(a) shows the XRD patterns of the precursor particles prepared by membrane emulsification after heat treatment at 400, 500, and 600 °C in air. No peaks were observed in the XRD pattern of the sample heat-treated at 400 °C, indicating that the particles were not crystallized. However, the diffraction patterns attributed to LiCoPO_4_ were observed in samples heat-treated at 500 and 600 °C. The peak intensity is higher at higher heating temperatures, indicating that the higher the heating temperature, the higher the crystallinity. Since the precursor particles obtained in this study were heat treated in powder form without pelleting, no cracking or other problems occurred even when the heat temperature was varied. Fig. 3(b) shows Raman spectra of the samples after heating. In both samples heat-treated at 400 and 500 °C, a peak at 1590 cm^−1^ attributed to graphite (G-band) and a peak at 1350 cm^−1^ attributed to the defect structure of graphite (D-band) were observed, indicating that the polyacrylamide in the precursor particles was carbonized. On the other hand, in the sample heat-treated at 600 °C, no peaks were observed at the G- and D-band positions, indicating that the carbon was combusted from the fine particles during heat treatment at 600 °C in air.^30^ These results indicate that LiCoPO_4_ particles with carbon can be formed when the precursor particles obtained in this study are heat-treated at 500 °C.

**Fig. 4:**
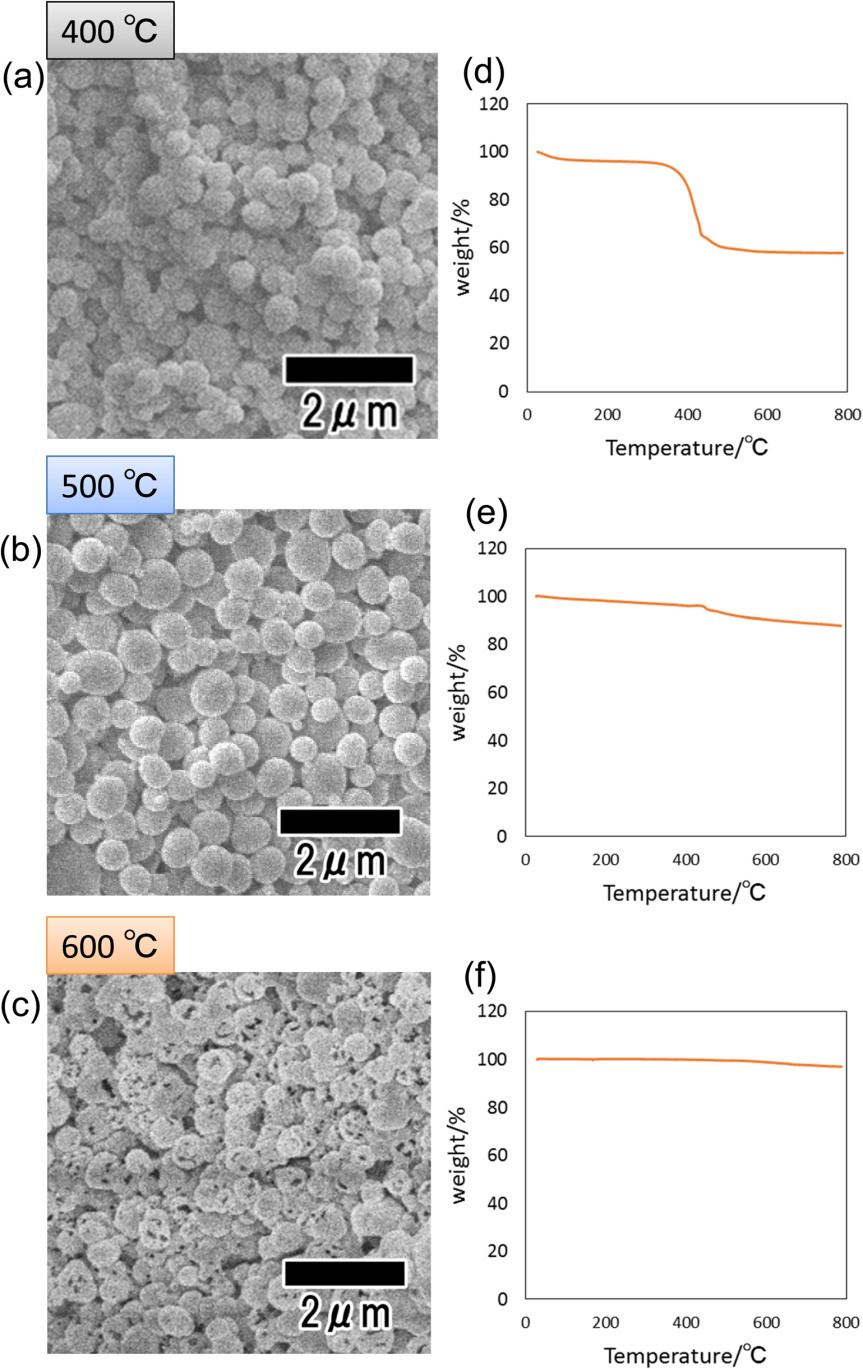
SEM images of particles after heat treatment at (a) 400, (b) 500, and (c) 600 °C. TG curves of particles after heat treatment at (d) 400, (e) 500, and (f) 600 °C.

Fig. 4(a)–(c) show SEM images of the particles heat-treated at 400, 500, and 600 °C. Spherical particles were observed in all samples, indicating that the shape of the precursor particles was retained even after heat treatment. However, the sample heat-treated at 600 °C, which showed no peak attributed to carbon in its Raman spectrum shown in Fig. 3(b), was confirmed to contain particles with a porous structure. We consider that this is due to the combustion of the polymer as well as the increase in crystal size and the formation of voids inside the particles. Fig. 4(d)–(f) show the results of thermal analysis to determine the amount of carbon content remaining inside the particles after heat treatment. Each sample was heat-treated at 400, 500, and 600 °C, then heat-treated again in the air from room temperature to 800 °C, and the weight change was measured. In this experiment, the weight loss due to the reheating treatment of samples is considered to originate from the carbon component of the fine particles because all carbon components are combusted in air under high-temperature conditions. From Fig. 4(d), a large weight loss was observed at around 410 °C in the sample heat-treated at 400 °C. The weight loss was 40 wt% of the initial weight, confirming the presence of 40 wt% carbon inside the particles heat-treated at 400 °C. On the other hand, the particles heat-treated at 500 and 600 °C showed weight losses of 12 and 2 wt%, respectively. These results indicate that the higher the heat treatment temperature, the more carbon is combusted and that no carbon remains in the sample heat-treated at 600 °C or higher.

**Fig. 5:**
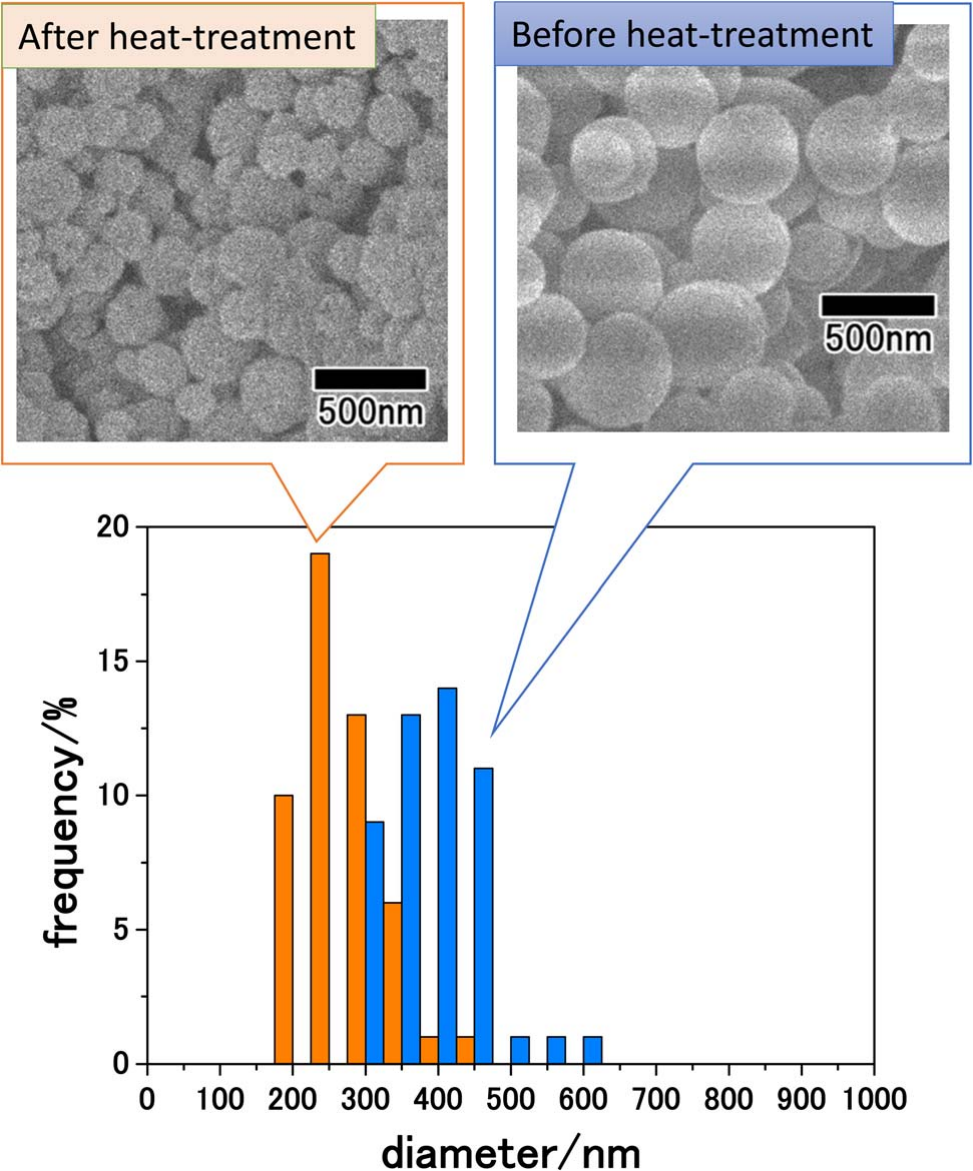
SEM images and size distribution histograms of LiCoPO_4_ particles before and after heat treatment at 500 °C.

Fig. 5 shows SEM images of the precursor particles obtained by membrane emulsification and LiCoPO_4_ particles after heat treatment at 500 °C, as well as the size distribution of each particle. SEM images show that although both precursor and LiCoPO_4_ particles are spherical, their sizes are reduced by heat treatment. The size distribution obtained by measuring the sizes of 300 particles from SEM images confirmed that both types of particle have a peak with a small distribution width. The average sizes of the particles before and after heat treatment were 410 and 250 nm, respectively. The size reduction of the particles due to heat treatment is considered to be due to the fact that most of the polymers in the precursor particles were combusted during heat treatment.

**Fig. 6:**
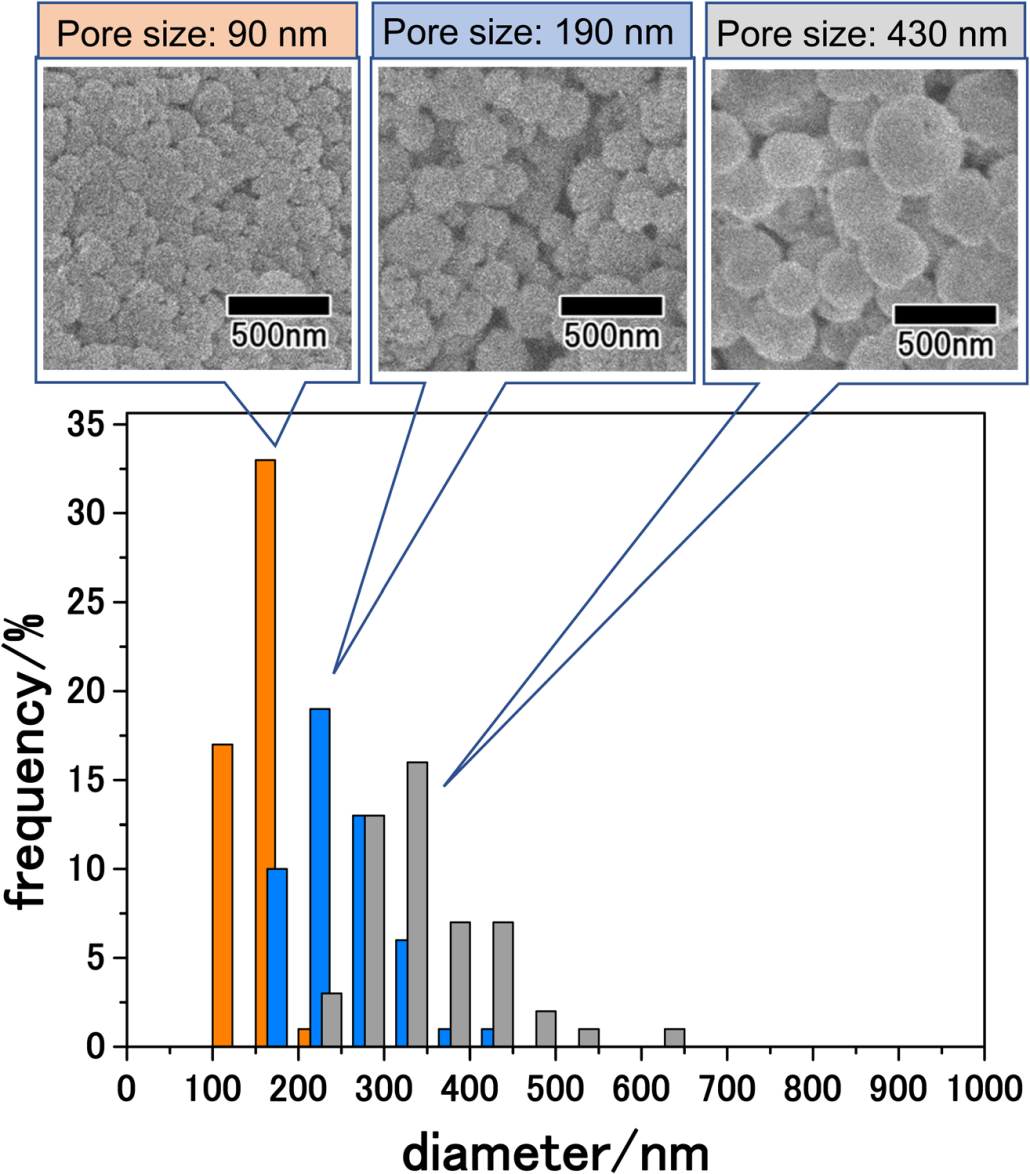
SEM images and size distribution histograms of LiCoPO_4_ particles prepared by membrane emulsification using anodic porous alumina with pore sizes of 90, 190, and 430 nm. The obtained precursor particles were heat-treated at 500 °C.

The size of particles obtained by membrane emulsification can be controlled by changing the pore size of emulsification membranes. The pore size of anodic porous alumina can be adjusted to 30–90% of the interpore distance by pore-widening treatment. Since the interpore distance of anodic porous alumina can be controlled by changing the anodization voltage, the pore size can be controlled in the range of 10 nm to 1 μm by combining changes in anodization voltage and pore-widening treatment. Fig. 6 shows the results for the LiCoPO_4_ particles obtained by membrane emulsification using anodic porous alumina with pore sizes of 90, 190, and 430 nm. The anodic porous alumina with a pore size of 90 nm was prepared by anodization of Al at 120 V for 20 h in a mixture of 0.25 M oxalic acid and 0.3 M phosphoric acid. The anodic porous alumina with a pore size of 430 nm was also prepared by Al anodization at 300 V for 6 h in a mixture of 0.2 M citric acid and 2 mM phosphoric acid. For both samples, the alumina membranes were detached by two-layer anodization using 17.6 M sulfuric acid. All the particles shown in Fig. 6 were observed after heat treatment at 500 °C. The SEM images in Fig. 6 show that the smaller the pore size of the alumina membrane used for membrane emulsification, the smaller the size of the resulting LiCoPO_4_ particles. Form the histogram of the particle size distribution shown in Fig. 6, the size distribution obtained for LiCoPO_4_ particles through this method is smaller than the one obtained conventionally by other reported methods.^31,32^ In the histograms, although there were differences in peak height, the relative standard deviation, which indicates size variation, was similar for all samples at *ca.* 20%. The average sizes of the particles prepared using alumina membranes with pore sizes of 90, 190, and 430 nm were 160, 250, and 340 nm, respectively.

### Electrochemical properties of obtained LiCoPO_4_ particles

**Fig. 7:**
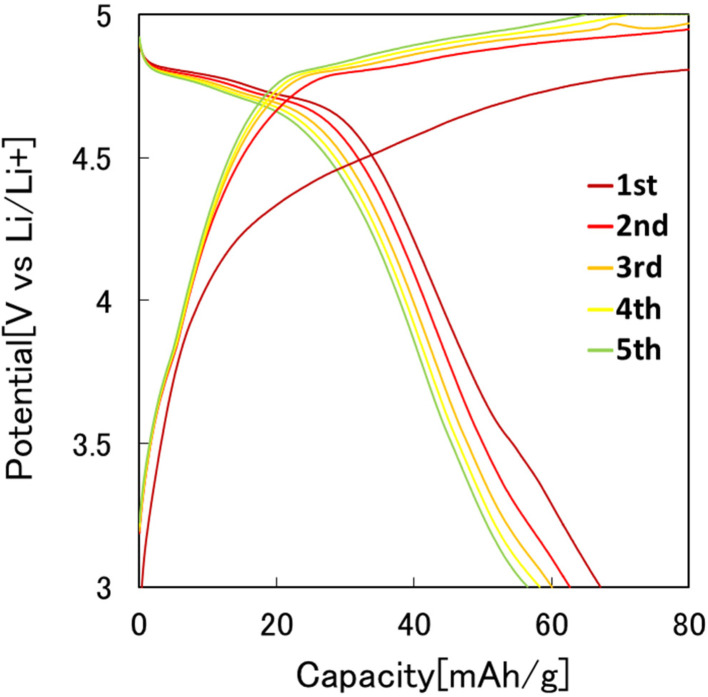
Charge–discharge curves at 0.1C rate of LiCoPO_4_ particles prepared by present process.

Fig. 7 shows the results of the constant-current charge–discharge test using the 2032 coin-type cell fabricated with the LiCoPO_4_ particles obtained in this study as the cathode active materials. In this experiment, LiCoPO_4_ particles containing carbon with an average diameter of 250 nm, obtained by the heat treatment of the precursor particles at 500 °C, were used. From the charge–discharge curve shown in Fig. 7, it was observed that the first discharge capacity was 67 mA h g^−1^. This result indicates that the LiCoPO_4_ particles obtained in this study can function as the cathode active materials for LIBs. The first discharge capacity of the LiCoPO_4_ particles prepared in this study was lower than the previous reported results.^33^ The reason for this is not clear at this stage and will be clarified in future studies.

**Fig. 8:**
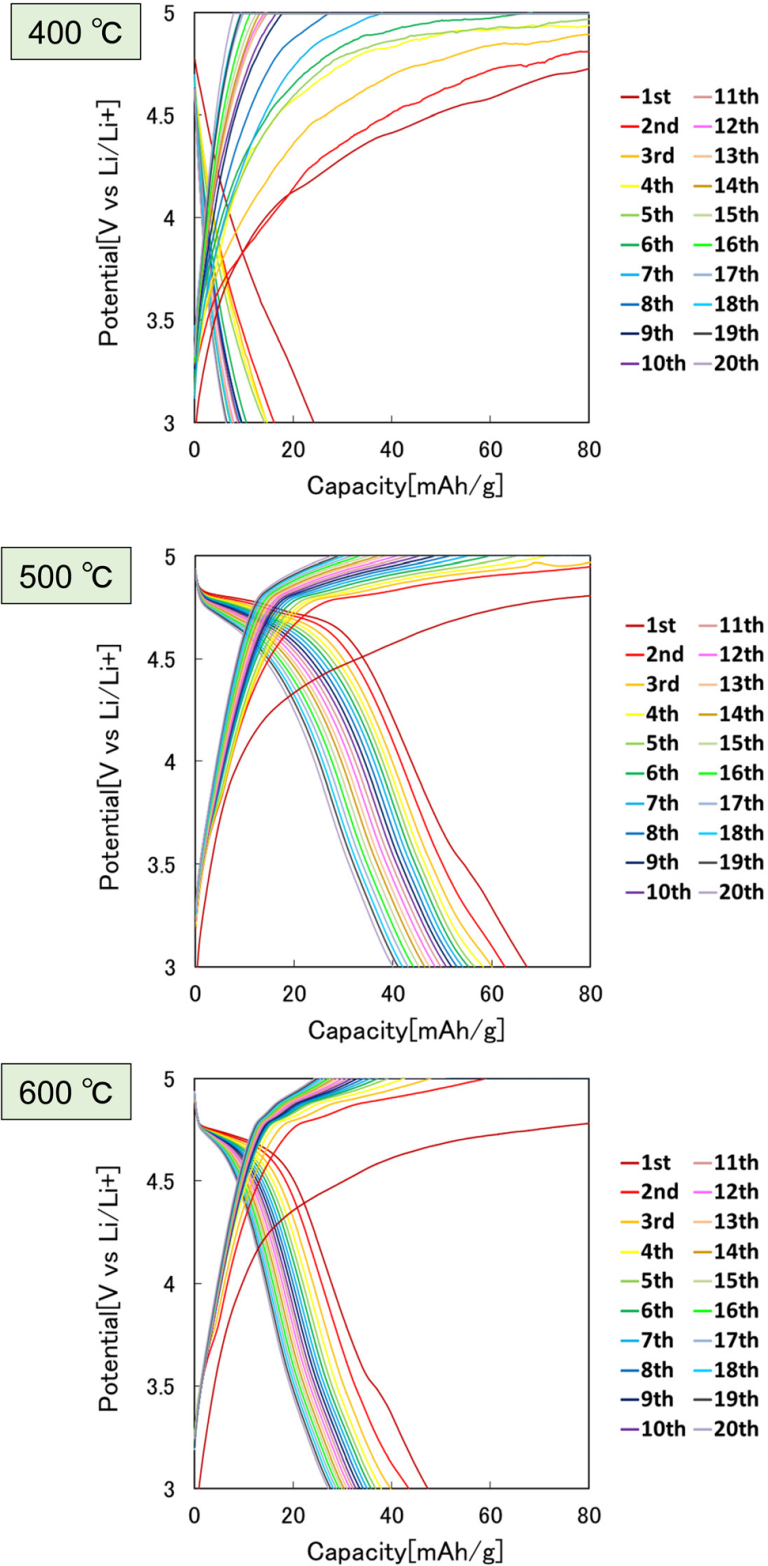
Charge–discharge curves at 0.1C rate of LiCoPO_4_ particles prepared by heat treatment of precursor particles at 400, 500, and 600 °C.

Fig. 8 shows the effect of the heat treatment temperature of the precursor particles on the cathode properties. In this experiment, the particles heat-treated at 400, 500, and 600 °C were used as the cathode active materials for LIBs. From the charge–discharge curves shown in Fig. 8, the highest discharge capacity was observed for the particles heat-treated at 500 °C. The sample heat-treated at 400 °C had low crystallinity, and the sample heat-treated at 600 °C had low electric conductivity due to the lack of carbon, resulting in poor cathode properties.

**Fig. 9:**
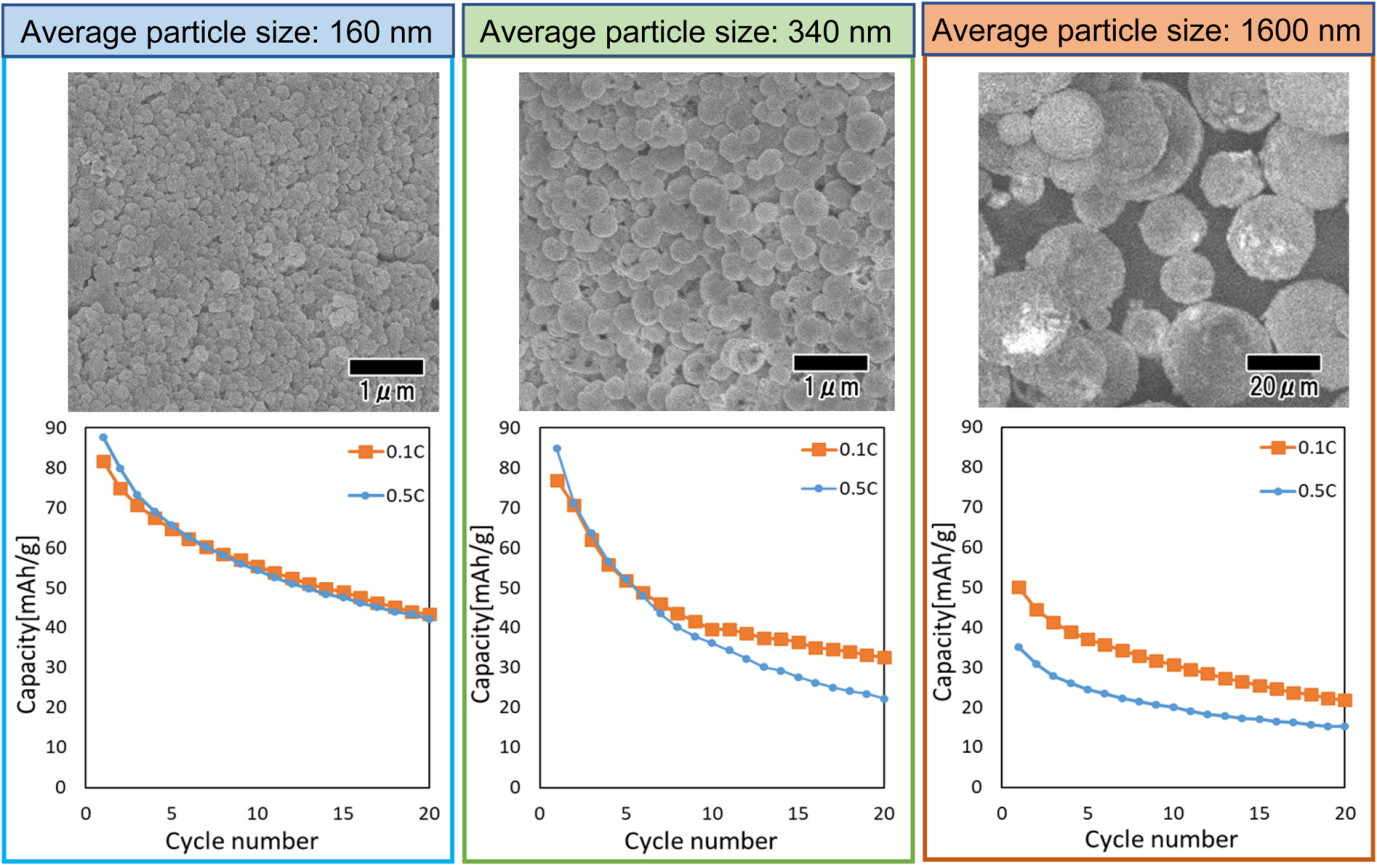
SEM images and cycle performance characteristics of LiCoPO_4_ particles with sizes of 160, 340, and 1600 nm.

Fig. 9 shows the results of a study of the effect of particle size on cathode properties. In this study, LiCoPO_4_ particles with average sizes of 160, 340, and 1600 nm were used. The LiCoPO_4_ particles with average sizes of 160 and 340 nm were prepared by membrane emulsification using anodic porous alumina, and the particles with an average size of 1600 nm were prepared by emulsification with stirring with a magnetic stirrer. All particles were prepared under the same conditions using the same raw materials, except for the emulsification method. The heat treatment temperature was set to 500 °C, which showed the best result in the experiment as shown in Fig. 8. The SEM images shown in Fig. 9 indicate that all particles are a spherical. The graphs in Fig. 9 summarize the cycle characteristics of discharge capacity when charge–discharge tests at 0.1 and 0.5C rates were conducted using each type of particle. The discharge capacity of all samples decreased as the cycles progressed. In addition, the smaller the particle size, the smaller the degree of decrease in discharge capacity. LiCoPO_4_ particles with an average diameter of 160 nm, the smallest particles used in this experiment, exhibited similar cycle characteristics in charge–discharge tests at both 0.1 and 0.5C rates. On the other hand, for LiCoPO_4_ particles larger than 340 nm, it was observed that the cycle characteristic degradation was greater in the charge–discharge test at 0.5C rate than in the charge–discharge test at 0.1C rate. This result indicates that as the particles become smaller, the Li-ion diffusion length in the active material becomes smaller, resulting in lower level of degradation of cycle characteristics even when the C rate during the charge–discharge test is increased. Further improvement of charge–discharge characteristics is expected in the future by reducing the particle size and optimizing the heat treatment conditions.

## Conclusions

Membrane emulsification using anodic porous alumina enabled the preparation of precursor particles composed of a polymer containing Li and Co salts. Spherical LiCoPO_4_ particles with uniform sizes were obtained by the heat treatment of the resulting precursor particles. Composite particles of LiCoPO_4_ and carbon were also obtained by adjusting the heat treatment temperature.

These results indicate that it is possible to prepare crystalline and conductive LiCoPO_4_ particles by optimizing the heat treatment temperature. The size of the LiCoPO_4_ particles obtained by this process could be controlled arbitrarily by changing the pore size of the porous alumina membrane used for emulsification. From the results of electrochemical measurements, the obtained LiCoPO_4_ particles were effective as cathode active materials for LIBs, and the cycle properties were observed to improve as the particle size was reduced. This result indicates that as the particles become smaller, the Li-ion diffusion length in the active material becomes smaller, resulting in lower level of degradation of cycle characteristics. In this report, we described the preparation of LiCoPO_4_ particles by membrane emulsification using anodic porous alumina and their application to LIBs. This method is useful for the preparation of size-controlled particles composed of various metal oxides since the size-controlled particles are formed by membrane emulsification using an aqueous solution containing a water-soluble monomer and metal salts as the dispersed phase, followed by heat treatment. The size-controlled particles obtained by this process are expected to be used not only as cathode active materials for LIBs, but also as component of various other applications, such as sensors, catalysts, and drug carriers.

## Author contributions

Takashi Yanagishita: conceptualization, methodology, writing-original draft. Raraka Otomo: investigation. Hideki Masuda: supervision, writing-review & editing.

## Conflicts of interest

There are no conflicts to declare.

## Supplementary Material
